# Supplementary material for: Phenotypic and Genomic Difference among Four Botryosphaeria Pathogens in Chinese Hickory Trunk Canker
Source: J Fungi (Basel). 2023 Feb 4;9(2):204. doi: 10.3390/jof9020204 (PMC9963396; doi:10.3390/jof9020204)
Supplement: Supplementary file 1 [file jof-09-00204-s001.zip › Supplementary Table S3.pdf]

Supplementary Table S3 Specific candidate primers for *B. dothidea* molecular identification based on species-specific genes.

Number	Gene	Gene_Length(bp)	Primer				Product size(bp)
1	*jg10975*	942	Sense	5'	TCTGGTGCTCCACATCGC	3'	650
			Anti-sense	5'	CGCATAGTCATCGCCCTC	3'	
3	*jg10954*	1425	Sense	5'	CGATGAGGTGAGCGATGC	3'	580
			Anti-sense	5'	CGCGATGTGCGTTATGGA	3'	
4	*jg10969*	955	Sense	5'	GACGCCCGATCTTTCTCA	3'	689
			Anti-sense	5'	GCTGGTCGTTGCCTCTGT	3'	
6	*jg1156*	938	Sense	5'	ATTCCGCAACCCCACTTC	3'	784
			Anti-sense	5'	TCCCCACAACTACTGTCTGA	3'	
7	*jg1162*	754	Sense	5'	GGCAGAGGTGGGTTTGAG	3'	572
			Anti-sense	5'	AGGGTGCGGATGCGATAT	3'	
8	*jg10944*	1077	Sense	5'	CTCACCTTCCACATCAACCC	3'	667
			Anti-sense	5'	ACCGGCGCAGGCTCCTCTT	3'	
9	*jg10962*	915	Sense	5'	ACAACACCATCGCTCCCG	3'	665
			Anti-sense	5'	TTCGCACATATCACCTCCA	3'	
10	*jg11*	921	Sense	5'	TCCAACGACGAGCAATCC	3'	695
			Anti-sense	5'	TGTGCCCTGAGGCGGTAT	3'	
